# Body mass index is associated with epigenetic age acceleration in the visceral adipose tissue of subjects with severe obesity

**DOI:** 10.1186/s13148-019-0754-6

**Published:** 2019-12-02

**Authors:** Juan de Toro-Martín, Frédéric Guénard, André Tchernof, Frédéric-Simon Hould, Stéfane Lebel, François Julien, Simon Marceau, Marie-Claude Vohl

**Affiliations:** 1 Institute of Nutrition and Functional Foods (INAF), Université Laval, Pavillon des Services (2729 K), 2440, boul. Hochelaga, Quebec, QC G1V 0A6 Canada; 2 School of Nutrition, Université Laval, Quebec, QC Canada; 3 Quebec Heart and Lung Institute Research Center, Quebec, QC Canada; 4 Department of Surgery, Université Laval, Quebec, QC Canada

**Keywords:** Epigenetic aging, Obesity, Visceral adipose tissue, Weight loss, Metabolic syndrome, Bariatric surgery, Epigenetic clock

## Abstract

**Background:**

There is solid evidence that obesity induces the acceleration of liver epigenetic aging. However, unlike easily accessible blood or subcutaneous adipose tissue, little is known about the impact of obesity on epigenetic aging of metabolically active visceral adipose tissue (VAT). Herein, we aimed to test whether obesity accelerates VAT epigenetic aging in subjects with severe obesity.

**Results:**

A significant and positive correlation between chronological age and epigenetic age, estimated with a reduced version of the Horvath’s epigenetic clock, was found in both blood (*r* = 0.78, *p* = 9.4 × 10^−12^) and VAT (*r* = 0.80, *p* = 1.1 × 10^−12^). Epigenetic age acceleration, defined as the residual resulting from regressing epigenetic age on chronological age, was significantly correlated with body mass index (BMI) in VAT (*r* = 0.29, *p* = 0.037). Multivariate linear regression analysis showed that, after adjusting for chronological age, sex and metabolic syndrome status, BMI remained significantly associated with epigenetic age acceleration in VAT (beta = 0.15, *p* = 0.035), equivalent to 2.3 years for each 10 BMI units. Binomial logistic regression showed that BMI-adjusted epigenetic age acceleration in VAT was significantly associated with a higher loss of excess body weight following biliopancreatic diversion with duodenal switch surgery (odds ratio = 1.21; 95% CI = 1.04–1.48; *p* = 0.03).

**Conclusions:**

Epigenetic age acceleration increases with BMI in VAT, but not in blood, as previously reported in liver. These results suggest that obesity is associated with epigenetic age acceleration of metabolically active tissues. Further studies that deepen the physiological relevance of VAT epigenetic aging will help to better understand the onset of metabolic syndrome and weight loss dynamics following bariatric surgery.

## Background

Obesity is a condition with a complex and heterogenous metabolic phenotype [1, 2]. Apart from its main feature, an excessive body fat accumulation in body fat depots, a plethora of additional harmful metabolic disturbances may appear in patients affected by obesity [3]. Among these, increased fasting plasma triglyceride and glucose levels, reduced HDL-cholesterol and elevated blood pressure, together with increased waist circumference, represent a set criteria often used to identify patients with the metabolic syndrome [4]. The frequency and severity of these comorbidities varies widely among patients with obesity, raising questions about the onset of metabolically unhealthy phenotypes in certain patients [5]. Mounting evidence suggests that, in addition to body mass index (BMI), sex, age or body fat distribution, the prevalence of obesity comorbidities depends on the concurrence of multiple factors, among which genetics and epigenetics would be playing a prominent role [6, 7].

Focusing on epigenetics, we and others have shown that altered DNA methylation in obesity would be associated to increased prevalence of metabolic comorbidities [8–13]. As such, both global methylation differences observed in blood and tissue-specific methylation alterations have been found to be associated with healthy or unhealthy obesity phenotypes [14, 15]. Likewise, given the innate plasticity of DNA methylation at cytosine-phosphate-guanine (CpG) dinucleotides [16], its modulation appears to be mediated through numerous environmental and lifestyle factors, such as diet or metabolic stress, as well as by intrinsic individual features, mainly sex and age [17]. Under this multifactorial scenario, the concept of epigenetic aging emerges as a straightforward approach to illustrate how a complex environment such as obesity may impact the epigenetic signature of human tissues [18].

Methylation levels of a number of CpG sites positively correlate with chronological age [19, 20]. These and other findings led to the construction of various algorithms allowing to estimate epigenetic age, also known as DNA methylation age (DNAm age), a novel parameter able to accurately measure an individual’s age [21–23]. Epigenetic age acceleration, i.e. the deviation of epigenetic age from chronological age, given a specific metabolic condition then reflects the impact of such condition on epigenetic age. That is the case in obesity, where BMI has been found to be associated with epigenetic age acceleration [24]. Interestingly, the positive correlation between BMI and epigenetic age acceleration does occur only in liver, while no association is observed in blood or in other sites, such as muscle or subcutaneous adipose tissue [24].

Given the relevance of liver as a central regulator of metabolism under both physiological and pathological conditions, these results point out to a specific impact of obesity on the epigenetic aging of metabolically active tissues [25]. These findings gain significance when considering that the acceleration of epigenetic age in visceral adipose tissue (VAT), a key tissue in obesity development and progression, has never been analyzed, probably due to its inaccessibility, as compared to more accessible blood or subcutaneous adipose tissue. Under this perspective, epigenetic aging of target tissues may also have an impact on the onset of metabolic syndrome and participate in other major metabolic processes occurring in obesity, such as body weight loss. We then hypothesized that, mirroring liver, BMI is associated with epigenetic age acceleration of VAT, which may explain part of the heterogeneity of obesity phenotypes and/or play a role in the interindividual variability previously observed in weight loss dynamics following bariatric surgery [26].

## Results

### Phenotype data distribution

After patient exclusions, 24 men and 28 women matched for age, BMI and metabolic syndrome were available for epigenetic aging analysis. Phenotype data passed normality tests. On the one hand, BMI ranged from 40.1 to 81.2 kg/m^2^ and was significantly higher in men than in women (mean = 54.4, SD = 9.1 vs mean = 48.9, SD = 7.2 kg/m^2^; *p* = 0.02) (Table 1). The replication liver dataset showed a wider BMI range (17.4–70.2 kg/m^2^), with women having a BMI higher than men (mean = 43.7, SD = 12.3 vs mean = 35.9, SD = 14.4 kg/m^2^; *p* = 0.04) (Table 1). BMI range in the validation liver subset including only subjects with severe obesity was closer to ours (40.4–70.2 kg/m^2^) and no difference was observed between men and women (Table 1). On the other hand, chronological age ranged from 18.8 to 54.4 years old in the entire population and no difference was found between men and women (Table 1). By contrast, women in the liver replication dataset, which ranged from 23.0 to 83.0 years old, were significantly younger than men (mean = 45.2, SD = 10.3 vs mean = 55.6, SD = 17.3; *p* = 0.01). Such a difference faded out in the subset of individuals with obesity (Table 1). As expected, no significant differences in BMI or chronological age were found within each sex group between matched individuals with and without metabolic syndrome (Additional file 1: Table S1).

**Table 1:** Data summary of the cohorts used in this study

Variable	Study participants (*n* = 52)					Liver (*n* = 62)					Liver (obesity) (*n* = 40)				
	Men		Women		*p*	Men		Women		*p*	Men		Women		*p*
	(*n* = 24)		(*n* = 28)			(*n* = 17)		(*n* = 45)			(*n* = 6)		(*n* = 34)		
	Mean	SD	Mean	SD		Mean	SD	Mean	SD		Mean	SD	Mean	SD	
Chronological age	35.0	10.6	32.9	5.7	0.36	55.6	17.3	45.2	10.3	0.01	51.2	10.0	43.6	8.9	0.07
BMI	54.4	9.1	48.9	7.2	0.02	35.9	14.4	43.7	12.3	0.04	54.1	4.7	49.5	6.8	0.12

Data are expressed as mean and standard deviation (SD). Liver and liver (obesity) refer to publicly available data (GSE48325) [27] and a subset of subjects with BMI > 40 kg/m^2^, respectively. *p* stands for *p* values obtained in Student’s *t* test. *BMI* body mass index

### Epigenetic age acceleration of VAT correlates with BMI

**Fig. 1:**
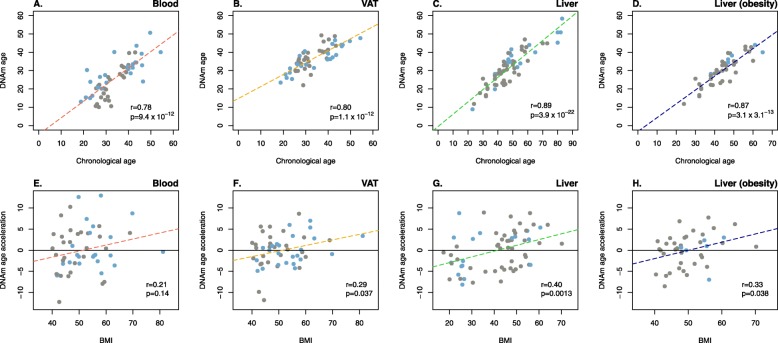
The acceleration of epigenetic aging in VAT correlates with BMI. First row of panels represents the correlation between chronological age and DNA methylation (DNAm) age, estimated with the reduced version of Horvath’s epigenetic clock [21], in blood (**a**, red dashed line), visceral adipose tissue—VAT—(**b**, yellow dashed line), liver (**c**, green dashed line), and in the liver of subjects with severe obesity (**d**, blue dashed line). Second row of panels from **e** to **h** show the correlation between residuals from regressing DNAm age on chronological age, and body mass index (BMI) in blood (**e**), VAT (**f**), liver (**g**), and in the liver of subjects with obesity (**h**). Residuals above zero (horizontal black line) stand for an acceleration of DNAm age. *r* and *p* stand for Pearson correlation coefficients and *p* values, respectively. Blue and gray dots refer to men and women, respectively

A strongly significant and positive correlation between chronological age and epigenetic age was found in both blood (*r* = 0.78, *p* = 9.4 × 10^−12^) (Fig. 1a) and VAT (*r* = 0.80, *p* = 1.1 × 10^−12^) (Fig. 1b). These results were similar to those previously obtained in liver [24], and successfully replicated herein with our reduced version of the epigenetic clock (*r* = 0.89, *p* = 3.9 × 10^−22^) (Fig. 1c), as well as to those in the liver of subjects with obesity (*r* = 0.87, *p* = 3.3 × 10^−13^) (Fig. 1d). Residuals resulting from regressing epigenetic age on chronological age were then used as a measurement of epigenetic age acceleration, whose association with BMI was tested. Epigenetic age acceleration in blood was not correlated with BMI (*r* = 0.21, *p* = 0.14) (Fig. 1e), as previously reported [24]. In contrast, a significant and positive correlation was found between epigenetic age acceleration and BMI in VAT (*r* = 0.29, *p* = 0.037) (Fig. 1f). A positive correlation was also observed in liver (*r* = 0.40, *p* = 0.0013) (Fig. 1g), where we were able to consistently reproduce previously reported experimental findings [24]. Results in the validation dataset showed that BMI correlated with epigenetic age acceleration in the liver of subjects with severe obesity to a similar extent to what we observed in VAT (*r* = 0.33, *p* = 0.038) (Fig. 1h). With the entire population of 52 individuals, a medium Pearson correlation coefficient of 0.38 is needed to attain a statistical power of 0.8. We also analyzed the impact of BMI on epigenetic age acceleration after excluding younger (10th percentile, ~ 24 years old) and older (90th percentile, ~ 44 years old) participants. The new results in the so-called middle-age dataset (*n* = 42, 14 men and 28 women) still showed a significant and even stronger correlation between BMI and epigenetic age acceleration in VAT (*r* = 0.34, *p* = 0.028), whereas results in blood revealed a non-significant and low correlation (*r* = 0.036, *p* = 0.82) (Additional file 1: Figure S1).

### BMI is consistently associated with epigenetic age acceleration of VAT

Multivariate linear regression models, adjusted by chronological age, sex and metabolic syndrome, confirmed that epigenetic age acceleration was not dependent on BMI in blood (beta = 0.16; 95% CI = − 0.04–0.36; *p* = 0.12) (Table 2). Our results also showed that BMI remained significantly associated with epigenetic age acceleration in VAT (beta = 0.15; 95% CI = 0.04–0.28; *p* = 0.03) (Table 2), as well as in liver (beta = 0.16; 95% CI = 0.07–0.25; *p* = 5.6 × 10^−4^) and in the liver of subjects with obesity (beta = 0.24; 95% CI = 0.04–0.45; *p* = 0.02). With beta estimates representing the acceleration of epigenetic age in years by unit change of BMI and after correcting for chronological age, the observed epigenetic age acceleration in VAT was equivalent to 2.20 years for each 10 BMI units, similar to the additional 2.28 years observed in liver, and the 3.04 years in the liver of subjects with obesity (Table 2).

**Table 2:** Association of BMI with epigenetic age acceleration in blood, VAT and liver

Variable	Blood				Visceral adipose tissue				Liver				Liver (obesity)		
	*β*	95% CI	*p*		*β*	95% CI	*p*		*β*	95% CI	*p*		*β*	95% CI	*p*
Chronological age	0.87	0.67–1.07	1.48 × 10^−11^		0.66	0.53–0.80	2.63 × 10^−13^		0.71	0.62–0.81	< 2 × 10^−16^		0.80	0.65–0.95	7.56 × 10^−13^
BMI	0.16	− 0.04–0.36	0.12		0.15	0.04–0.28	0.03		0.16	0.07–0.25	5.6 × 10^−4^		0.24	0.04–0.45	0.02
Sex	− 3.11	− 6.54–0.33	0.08		2.89	0.59–5.20	0.02		− 1.47	− 4.07–1.13	0.26		− 0.28	− 4.12–3.56	0.88
Metabolic syndrome	0.47	− 2.75–3.69	0.77		1.42	− 0.74–3.58	0.19		-	-	-		-	-	-
Adjusted *R*^*2*^	0.64				0.67				0.82				0.78		
Age acceleration	1.83				2.20				2.28				3.04		

Estimates (*β*), 95% confidence intervals (95%CI), and *p* values (*p*) are from multivariate linear regression models of DNA methylation age acceleration adjusted by chronological age, body mass index (BMI), sex and metabolic syndrome. *β* represents the acceleration of epigenetic age in years by unit change of dependent variable. Age acceleration stands for the increase of epigenetic age in years for each 10-point increase in BMI

### Epigenetic age acceleration of VAT correlates with BMI only in men

**Fig. 2:**
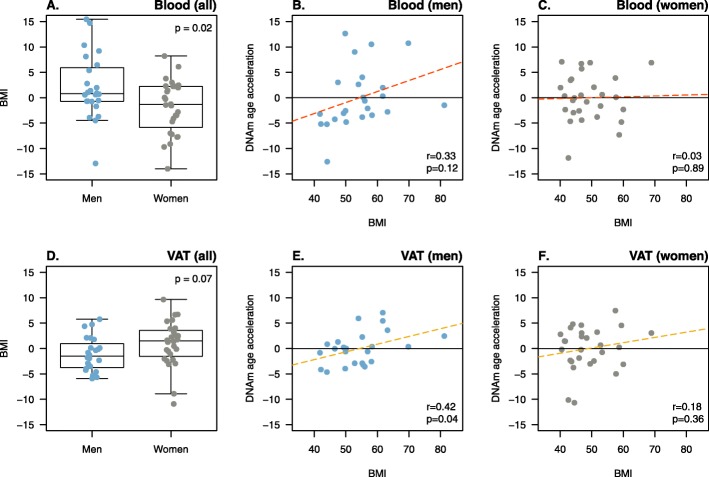
The acceleration of epigenetic aging in VAT correlates with BMI in men. Panels **a** and **d** compare the distribution of DNA methylation (DNAm) age acceleration, defined as the residual from regressing DNAm age on chronological age, between men and women in blood (**a**) and visceral adipose tissue—VAT—(**d**). Boxplots represent the distribution of phenotype data with median (dark horizontal line) and interquartile range (box), and *p* stands for the *p* values obtained in Student’s *t* test for independent samples. The correlation between DNAm age acceleration and body mass index (BMI) in blood (**b** and **c**; red dashed line) and VAT (**e** and **f**, yellow dashed line) is shown separately in men and women. Residuals over zero (horizontal black line) stand for an acceleration of DNAm age, while *r* and *p* refer to Pearson correlation coefficients and *p* values, respectively. Blue and gray dots refer to men and women, respectively.

In view that results from multivariate regression models showed a significant association between sex and epigenetic age acceleration (Table 2), the latter was compared between men and women, and its correlation with BMI was analyzed separately. Significant sex differences were observed in blood, with men having a higher epigenetic age acceleration than women (2.1 vs − 1.8, *p* = 0.02) (Fig. 2a). However, BMI was not correlated with epigenetic age acceleration in blood, neither in men (*r* = 0.33, *p* = 0.12) (Fig. 2b) nor in women (*r* = 0.03, *p* = 0.89) (Fig. 2c). Although no difference was found in epigenetic age acceleration between men and women in VAT (− 1.11 vs 0.95, *p* = 0.07) (Fig. 2d), BMI was significantly and positively correlated with epigenetic age acceleration in men (*r* = 0.42, *p* = 0.04) (Fig. 2c), but not in women (*r* = 0.19, *p* = 0.35) (Fig. 2d). With a population of 24 men and 28 women, Pearson correlation coefficients larger than 0.5 are needed to attain a statistical power of 0.8. By contrast, a significant correlation between BMI and liver epigenetic age acceleration was observed only in women (*r* = 0.45, *p* = 0.002), but not in men (*r* = 0.38, *p* = 0.13). Similar results were found in the liver obesity group in both women (*r* = 0.42, *p* = 0.013) and men (*r* = − 0.05, *p* = 0.93) (Additional file 1: Figure S2).

### Epigenetic age acceleration of VAT is associated with postsurgery weight loss

**Fig. 3:**
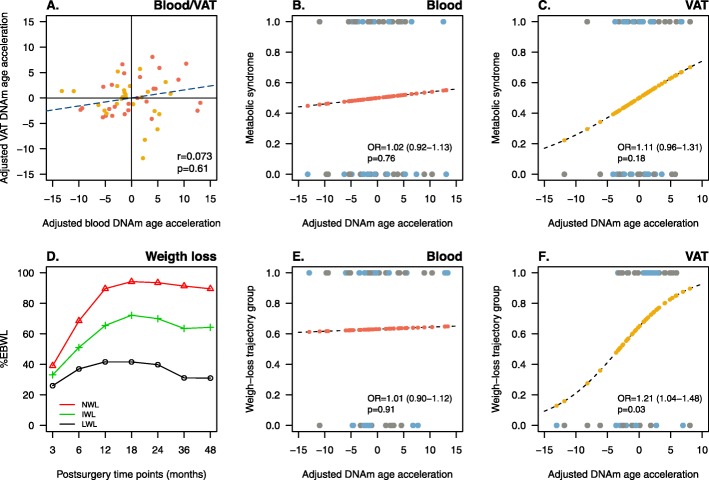
The acceleration of epigenetic aging in VAT is associated with a more pronounced weight loss response following bariatric surgery. Panel **a** shows the correlation between DNA methylation (DNAm) age acceleration adjusted by sex and body mass index (BMI) between blood and visceral adipose tissue—VAT. Red and yellow dots represent participants with and without metabolic syndrome. *r* and *p* refer to Pearson correlation coefficients and *p* values, respectively. Panels **b** and **c** show the predicted probability (from 0 to 1), obtained by binomial logistic regression, of each participant to have a healthy (0) or an unhealthy (1) obesity phenotype based on its adjusted DNAm age acceleration in blood (red dots, **b**) and VAT (yellow dots, **c**). Panel **d** shows the three weight-loss clusters resulting from estimating postsurgery excess body weight loss (%EBWL) trajectories for each participant over a follow-up period of 4 years in the *traj* R package. Red, green, and black lines stand for normal (NWL), intermediate (IWL) and low (LWL) weight-loss trajectory clusters, representing 65%, 30%, and 5% of participants. IWL and LWL clusters were reassigned into a unique group (ILWL). Panels **e** and **f** show the predicted probability (from 0 to 1), obtained by binomial logistic regression, of each participant to belongs to the NWL group (1) or to the ILWL group (0), based on its adjusted DNAm age acceleration in blood (red dots, **e**) and VAT (yellow dots, **f**). OR is the odds ratio with 95% confidence intervals and *p* is the *p* value for the linear trend of association. Blue and gray dots refer to men and women, respectively

The measurement of epigenetic age acceleration adjusted by chronological age, sex and BMI in blood and VAT was not significantly correlated with each other (Fig. 3a). Binomial logistic regression was further used to test whether adjusted epigenetic age acceleration was associated with metabolic syndrome and/or with weight loss trajectory groups. On the one hand, results from the linear trend test did not show an association between the adjusted epigenetic age acceleration with metabolic syndrome, neither in blood (OR = 1.02; 95% CI = 0.92–1.13; *p* = 0.76) (Fig. 3b) nor in VAT (OR = 1.11; 95% CI =0.96–1.31; *p* = 0.18) (Fig. 3c). On the other hand, weight loss clustering procedure resulted in three trajectory groups depending on the percentage of excess body weight loss (%EBWL) as follows: normal weight loss (NWL), intermediate weight loss (IWL), and low weight loss (LWL), representing 65%, 30%, and 5% of patients (Fig. 3d). Patients from IWL and LWL groups were reassigned into a unique group (ILWL). While no association was found in blood (OR = 1.01; 95% CI = 0.90–1.12; *p* = 0.91) (Fig. 3e), the probability of belonging to the NWL group significantly increased with the adjusted epigenetic age acceleration of VAT (OR = 1.21; 95% CI = 1.04–1.48; *p* = 0.03) (Fig. 3f). In other words, patients showing higher epigenetic age acceleration in VAT exhibited a more pronounced weight loss response to bariatric surgery. Whether the adjusted epigenetic age acceleration was associated with metabolic syndrome and/or with weight loss trajectory groups was further tested separately in men and women with no significant result (data not shown).

## Discussion

This is, to our knowledge, the first study to show the association between BMI and epigenetic age acceleration of VAT. Concretely, the main finding of the present work revealed that increasing BMI in obesity is positively correlated with epigenetic age acceleration in VAT. Importantly, this study also replicates the absence of association between BMI and epigenetic age acceleration in blood [24]. The present results also revealed that VAT epigenetic aging is more strongly related to BMI in men, as compared to women. Additional findings suggested that VAT epigenetic age acceleration may not have a major association with features of the metabolic syndrome in obesity, but a potential and significant effect on the evolution of body weight loss following bariatric surgery.

Previously, Horvath et al. [24] carried out an elegant study to test whether obesity may increase the acceleration of tissue aging. The original version of the epigenetic clock [21], herein used in its reduced form, was utilized to test whether BMI correlated with epigenetic age acceleration in various tissues. Results were very enlightening since they suggested a tissue-specific effect of obesity on the acceleration of epigenetic aging. Concretely, BMI was highly correlated with accelerated liver aging, whereas no effect was observed in blood and, more importantly, nor in subcutaneous adipose tissue. Since fat depots are submitted to an important metabolic stress during weight gain, we found particularly interesting and paradoxical the fact that epigenetic aging of subcutaneous adipose tissue was not altered by increasing BMI, and we decided to investigate whether a distinct effect of obesity on VAT was taking place. We hypothesized that BMI relates to accelerated epigenetic aging of metabolically active tissues, such as VAT and liver, and that such accelerated aging may in part be responsible for the shifting from healthy to unhealthy obesity phenotypes. Although the latter part of the hypothesis was not fully supported by results, our findings are still of interest. In particular, our results support a specific impact of obesity on the epigenetic aging of key metabolic tissues. As mentioned above, the significant correlation showed herein between BMI and epigenetic age acceleration in VAT mirrored in some way that previously observed in liver [28]. Both the similar effect size of BMI on epigenetic age acceleration and the estimated epigenetic aging in years found in both VAT and liver further supported the hypothesis of a tissue-specific dysregulation of methylation [15, 29, 30].

Some methodological differences between the previous study in the liver and the present analysis need to be clearly pointed out. First, we had to use a reduced version of the Horvath’s clock due to technical constraints. However, the successful replication of previous results supported the use of our modified version of the epigenetic clock. Concretely, almost identical correlation coefficients between chronological and epigenetic age were observed in both studies, while correlation coefficients with BMI in liver showed the same magnitude and direction than in Horvath’s study [24]. Second, unlike in the liver study, all the patients from the present work had severe obesity, with or without associated metabolic disturbances, which somehow limited the BMI study range. Nevertheless, the correlation between BMI and epigenetic age acceleration in VAT and liver from patients with obesity was again highly similar, thus supporting the validity of this study. Second, the decision of matching pairs for age, BMI and metabolic syndrome within each sex further limited the spectrum of eligible participants, leading to a narrower range of age, as compared to the Horvath’s study. Both BMI and age ranges may influence the final results, as well as sex representation [28, 31]. Our results showed that men had higher epigenetic aging rates than women in blood, which has been previously linked to a lower morbidity but higher mortality in men than in women [28]. On the other hand, while the impact of BMI on liver epigenetic aging has been previously observed in men and women [24], herein association in VAT was seen only in men. Though, the correlation between BMI and epigenetic age acceleration in liver was only significant in women in the discovery dataset in [24]. Herein, we also observed a significant correlation between BMI and liver epigenetic age acceleration only in women, which may suggest a different impact of BMI on liver and VAT epigenetic aging between men and women. Although the present results did not show a significant association between the adjusted measurement of epigenetic age acceleration and the presence or absence of metabolic syndrome, these results still suggest a sex-specific impact of obesity on VAT epigenetic aging, which could explain, in part, the distinct development of obesity comorbidities between men and women, rather than waist circumference or visceral fat accumulation per se [32]. In any case, these results should be taken with caution, because the effect sizes observed, together with the number of samples from each sex, could lead to uncertain results. We acknowledged that this represents a limitation of the present study. Similarly, the level of statistical power achieved in the entire dataset does not allow us to derive statements regarding the association between BMI and epigenetic aging as consistent as desired. This fact, together with the heterogeneous and inconsistent results previously reported in blood [22, 24, 31], prevent us from ascertaining an actual lack of association between BMI and the epigenetic age acceleration in blood. In spite of reducing the total number of subjects, we tried to support our findings by reanalyzing blood and VAT samples in a dataset without extreme age values, as previously shown [31]. Interestingly, a stronger relationship between BMI and epigenetic age acceleration in VAT was observed, as well as the near absence of association in blood, supporting results observed in the entire dataset. Yet, further studies in larger cohorts designed to capture a broad BMI and age spectrum, as well as to attain a reasonable degree of statistical power, are still required to elucidate the actual impact of BMI on the epigenetic age acceleration in blood and VAT. Taken together, although the specific nature of our cohort somehow limits the number of participants, we consider it worth to be studied, since the findings drawn from its analysis add valuable insights to the discussion on the role of BMI on epigenetic aging.

As just mentioned, another relevant finding of the present work that deserves to be highlighted is the absence of association between VAT epigenetic aging and metabolic syndrome. This is important since it does not support the second term of the main hypothesis, that is, the development of obesity comorbidities through an acceleration of VAT epigenetic age. Among others, a potential explanation of this result is the complexity of the chosen composite endpoint, that is, the presence or absence of metabolic syndrome defined as the sum of a number of metabolic disturbances [4]. Another rather unexpected result was the positive association between the acceleration of VAT epigenetic aging and postsurgery weight loss trajectories, especially when BMI is negatively associated with the percentage of excess body weight loss following bariatric surgery [26]. However, these latter results may suggest that bariatric surgery would yield more beneficial outcomes in those patients with aged VAT. Previous studies have already reported a deep epigenetic remodeling after different weight loss interventions in adipose tissue [33, 34]. In view of that, these results could imply that a more intense methylation remodeling may be taking place in an aged VAT following bariatric surgery, leading to a tissue rejuvenation. Since our longitudinal weight-loss study is still not finished, it is not yet possible to have access to postsurgery VAT methylation data that would allow us to establish a causal relationship. It is also worth noting that this is a whole-tissue analysis and that knowing which cell type is responsible for the accelerated aging of VAT might help understand the link with the response to weight loss. In this regard, senescence of adipocytes is a hallmark of adipose tissue aging, and it would be expected that senescent mature adipocytes would mobilize less fat during weight loss [35]. However, the observed accelerated aging might be associated with senescence of another cell type which might impair lipid mobilization. This could be cells from the stromal vascular fraction involved in adipose tissue remodeling [36], e.g. immune cells or endothelial cells of the vascular bed which could reduce blood flow to the tissue [37]. Further studies are in process and will help understand the actual impact of bariatric surgery on VAT epigenetic aging.

## Conclusions

In conclusion, our results seem to corroborate that obesity accelerates epigenetic aging of metabolically active tissues, such as VAT and liver. Likewise, these results suggest that epigenetic age acceleration in blood does not correlate with BMI in obesity. Moreover, BMI seems to have a more pronounced effect on epigenetic age acceleration in men than in women. Finally, while not having an effect on metabolic syndrome development, the acceleration of VAT epigenetic aging seems to play an important role in weight loss dynamics following bariatric surgery.

## Methods

### Study participants

A total of 56 patients, 28 men and 28 women with severe obesity (BMI > 40 kg/m^2^) and undergoing bariatric surgery (biliopancreatic diversion with duodenal switch) at the Quebec Heart and Lung Institute, were selected to participate in the present study. Patients were matched within each sex for age, BMI and the presence or absence of metabolic syndrome. Omental samples (VAT) were obtained during the course of surgery and blood samples were collected preoperatively. The surgical protocol, blood and VAT sample collection, and the standardized procedures to measure anthropometric and metabolic parameters are described elsewhere [38]. Patients were diagnosed with the metabolic syndrome when three or more criteria of the National Cholesterol Education Program Adult Treatment Panel III guidelines were present [4]. Waist circumference, blood pressure, HDL-cholesterol, plasma triglycerides and fasting glucose levels were measured preoperatively and used to identify those with the metabolic syndrome. Severe obesity was defined as BMI > 40 kg/m^2^, calculated as weight in kilograms divided by height in meters squared. Due to the lack of accurate phenotype data, one patient and its matching pair were excluded from further analyses.

### Genome-wide DNA methylation analysis

Genomic DNA of the 56 study participants was extracted from 200 mg of VAT using the DNeasy Blood & Tissue kit (QIAGEN, Mississauga, Ontario, Canada) and isolated from the blood buffy coat using the GenElute™ Blood Genomic DNA kit (Sigma, St Louis, MO, USA). Following quantification of DNA using both NanoDrop Spectrophotometer (Thermo Scientific, Wilmington, DE, USA) and PicoGreen DNA methods, DNA (1 μg) was bisulfite converted and quantitative genome-wide methylation analysis was conducted using Infinium HumanMethylation450 (450k) and EPIC platforms (Illumina, San Diego, CA) interrogating over 485,000 and 850,000 CpG sites at single-nucleotide resolution, respectively. Methylation arrays were processed at the McGill University and Génome Québec Innovation Centre (Montreal, Canada) according to the manufacturer’s instructions (Illumina, San Diego, CA). Methylation data was preprocessed and normalized using the *minfi* R package [39]. Before background correction and normalization, the 450k and EPIC arrays were combined and integrated into a virtual 450k array, leaving 453,093 CpG sites for further statistical analyses. The single-sample Noob (*ssNoob*) method was the preferred normalization procedure, as previously recommended when integrating data from multiple Infinium methylation arrays [40]. Methylation levels (beta values; *β*) were estimated as the ratio of signal intensity of the methylated alleles to the sum of methylated and unmethylated intensity signals of the alleles (*β* value = C/(T+C)). The *β* values varied from 0 (no methylation) to 1 (100% methylation). The overall correlation across 453,093 CpG sites between 450 k and EPIC arrays was very high in blood and VAT (*r* = 0.992 in both tissues). One sample did not fulfill methylation quality control criteria and was excluded, together with its matching pair, from further analyses.

### Epigenetic clock

Epigenetic age was estimated for each patient in VAT and blood according to the Horvath’s epigenetic clock [21], currently the gold-standard for determining epigenetic age in humans [41]. Because part of methylation data was obtained from the EPIC array, which does not include the whole dataset of 353 CpG sites used to build the original epigenetic clock, we estimated epigenetic age by using a reduced version of it, as previously done [42]. The final dataset used to estimate epigenetic age consisted on a set of 336 CpG sites, 4.8% less than in the original dataset. The correlation among CpG sites included in the epigenetic clock was similarly strong in blood (*r* = 0.992) and VAT (*r* = 0.991) between 450k and EPIC arrays, with a total of 323 CpG sites in blood (96.1%) and 318 CpG sites in VAT (94.6%) showing a mean *β* value difference lower than 0.1 (Additional file 1: Figure S3). Aiming to analyze whether this modification could cause a lack of accuracy, we replicated previous results obtained with the original epigenetic clock in liver [24]. Publicly available methylation data from 62 liver samples (GSE48325) [27] was used as a replication dataset. A subset of 40 liver samples including only subjects with severe obesity (BMI > 40 kg/m^2^) was also used for comparison purposes. The reduced version of the epigenetic clock was built in R using publicly available data (https://horvath.genetics.ucla.edu/html/dnamage/) [21].

### Weight loss trajectories

Postsurgery weight loss trajectories were estimated for each participant by identifying clusters of individual longitudinal weight loss data implemented in the *traj* R package [43]. Briefly, the *traj* procedure uses a factor analysis to select nonredundant measurements, followed by a cluster analysis to identify subsets of patients with similar weight loss trajectories [44]. Weight loss data from 46 patients was available for trajectory group assessment. Body weight was measured during postoperative visits or phone calls thorough a follow-up period of four years, and a total of seven postsurgery time points at 3, 6, 12, 18, 24, 36, and 48 months were used for cluster estimation. Postsurgery weight loss was defined as ﻿the percentage of excess body weight loss (%EBWL), calculated as the difference between actual body weight loss (initial BMI minus actual BMI) and ideal body weight loss (initial BMI minus ideal BMI fixed at 25 kg/m^2^) [45]. Resulting groups allowed the categorization of patients as a function of their %EBWL.

### Statistics

Phenotype data was checked for normality with the Kolmogorov-Smirnov test. Two-group comparisons were tested with Student’s *t* test for paired and independent samples, as appropriate. Epigenetic age acceleration was defined as the residual resulting from regressing epigenetic age on chronological age, and its correlation with BMI was tested in both VAT and blood using Pearson correlation coefficients. A multivariate linear regression model including chronological age, sex and metabolic syndrome was further used to test the association between BMI and epigenetic age acceleration. Binomial logistic regression was used to predict the probability that a patient falls into a healthy or unhealthy phenotype (presence or absence of metabolic syndrome), as well as into a weight loss trajectory group, both set as dichotomous variables. Epigenetic age acceleration adjusted by chronological age, sex and BMI was set as a continuous variable in logistic regression. Regression models and the rest of statistical calculations were performed in R (https://www.R-project.org) [46]. Power calculations were performed in G*Power [47].

## Supplementary information

**Additional file 1: Table S1.** Data summary of participants in the study cohort. **Figure S1.** The acceleration of epigenetic aging in VAT correlates with BMI in middle-aged subjects. **Figure S2.** The acceleration of epigenetic age in liver correlates with BMI in women. **Figure S3.** Correlation among CpG sites included in the epigenetic clock between 450k and EPIC arrays.

## Data Availability

The datasets used and/or analyzed during the current study are available from the corresponding author on reasonable request.

## Abbreviations

%EBWL: Percentage of excess body weight loss
BMI: Body mass index
CpG: Cytosine-phosphate-guanine dinucleotides
DNAm age: DNA methylation age
IWL: Intermediate weight loss
LWL: Low weight loss
NWL: Normal weight loss
VAT: Visceral adipose tissue
