# Supplementary material for: Body mass index is associated with epigenetic age acceleration in the visceral adipose tissue of subjects with severe obesity
Source: Clin Epigenetics. 2019 Dec 2;11:172. doi: 10.1186/s13148-019-0754-6 (PMC6888904; doi:10.1186/s13148-019-0754-6)
Supplement: Supplementary file 1 — Additional file 1: Table S1. Data summary of participants in the study cohort. Figure S1. The acceleration of epigenetic aging in VAT correlates with BMI in middle-aged subjects. Figure S2. The acceleration of epigenetic age in liver correlates with BMI in women. Figure S3. Correlation among CpG sites included in the epigenetic clock between 450k and EPIC arrays. [file 13148_2019_754_MOESM1_ESM.docx]

**Title:** Body mass index is associated with epigenetic age acceleration in the visceral adipose tissue of subjects with severe obesity

Authors:

Juan de Toro-Martín^1,2^, juan.de-toro-martin.1@ulaval.ca

Frédéric Guénard^1,2^, Frederic.Guenard@fsaa.ulaval.ca

André Tchernof^2,3^, andre.tchernof@criucpq.ulaval.ca

Frédéric-Simon Hould^4^, Frederic.Hould@fmed.ulaval.ca

Stéfane Lebel^4^, stefane.lebel@fmed.ulaval.ca

François Julien^4^, francois.julien@criucpq.ulaval.ca

Simon Marceau^4^, simon.marceau@fmed.ulaval.ca

Marie-Claude Vohl^1,2^*, marie-claude.vohl@fsaa.ulaval.ca

^1^Institute of Nutrition and Functional Foods (INAF), Université Laval, Quebec, QC, Canada

^2^School of Nutrition, Université Laval, Quebec, QC, Canada

^3^Quebec Heart and Lung Institute Research Center, Quebec, QC, Canada

^4^Department of Surgery, Université Laval, Quebec, QC, Canada

***Corresponding author:**

Marie-Claude Vohl, PhD

Institute of Nutrition and Functional Foods (INAF)

Pavillon des Services (2729K)

2440, boul. Hochelaga

Université Laval

Quebec, QC, Canada

G1V 0A6

**Additional file 1**

**Index**

- **Table S1. ﻿Data summary of participants in the study cohort.**
- **Figure S1. The acceleration of epigenetic aging in VAT correlates with BMI in middle-aged subjects.**
- **Figure S2. The acceleration of epigenetic age in liver correlates with BMI in women.**
- **Figure S3. Correlation among CpG sites included in the epigenetic clock between 450k and EPIC arrays.**

**Table S1.** **﻿Data summary of participants in the study cohort.**

|  |  | Men (n=24) | | | | |  |  | Women (n=28) | | | | |  |
| --- | --- | --- | --- | --- | --- | --- | --- | --- | --- | --- | --- | --- | --- | --- |
|  |  | Healthy | |  | Unhealthy | |  |  | Healthy | |  | Unhealthy | |  |
|  |  | (n=12) | |  | (n=12) | |  |  | (n=14) | |  | (n=14) | |  |
| Variable |  | Mean | SD |  | Mean | SD | *P* |  | Mean | SD |  | Mean | SD | *P* |
| Chronological age | | 34.4 | 11.2 |  | 35.6 | 10.4 | 0.79 |  | 33.0 | 5.8 |  | 32.8 | 5.8 | 0.92 |
| BMI |  | 55.1 | 9.6 |  | 53.7 | 9.0 | 0.72 |  | 48.5 | 6.7 |  | 49.4 | 7.9 | 0.75 |

Data are expressed as mean and standard deviation (SD). Healthy and unhealthy refer to subjects without or with metabolic syndrome, respectively. Patients were diagnosed with the metabolic syndrome when three or more criteria of the National Cholesterol Education Program Adult Treatment Panel III guidelines were present. *P* stands for p-values obtained in Student’s t-test. BMI, body mass index.

**Figure S1.** **The acceleration of epigenetic aging in VAT correlates with BMI in middle-aged subjects.** Panels show the correlation between residuals from regressing DNA methylation (DNAm) age on chronological age, and body mass index (BMI) in blood (A, red dashed line) and visceral adipose tissue – VAT – (B, yellow dashed line) in middle-aged subjects (n=42). Residuals above zero (horizontal black line) stand for an acceleration of DNAm age. r and p stand for Pearson correlation coefficients and p-values, respectively. Blue and grey dots refer to men and women, respectively.

**Figure S2.** **The acceleration of epigenetic age in liver correlates with BMI in women.**

First row of panels represents the correlation between DNA methylation (DNAm) age acceleration and body mass index (BMI) in the liver of men (A) and women (B). Second row of panels represents the correlation between DNAm and BMI in the liver of men with obesity (C) and women with obesity (D), Residuals over zero (horizontal black line) stand for an acceleration of DNAm age, while r and p refer to Pearson correlation coefficients and p-values, respectively. Blue and grey dots refer to men and women, respectively. Green and blue dashed lines refer to liver and liver of subjects with obesity, respectively.

**Figure S3.** **Correlation among CpG sites included in the epigenetic clock between 450k and EPIC arrays.** Top row of panels represents the correlation among methylation levels of the 336 CpG sites included in the epigenetic clock between 450k (y axis) and EPIC (x axis) arrays in blood (A), and the frequency distribution of the mean β value difference among the 336 CpG methylation levels in blood (B). Bottom row of panels (C-D) shows the same data and in the same order as the top row but in visceral adipose tissue (VAT) samples. Blue and grey dots refer to men and women, respectively. Red and yellow dashed lines refer to blood and VAT samples, respectively.
